# ErbB4 Null Mice Display Altered Mesocorticolimbic and Nigrostriatal Dopamine Levels as well as Deficits in Cognitive and Motivational Behaviors

**DOI:** 10.1523/ENEURO.0395-19.2020

**Published:** 2020-05-21

**Authors:** Miguel Skirzewski, Marie E. Cronin, Ricardo Murphy, Wambura Fobbs, Alexxai V. Kravitz, Andres Buonanno

**Affiliations:** 1 Section on Molecular Neurobiology, Eunice Kennedy Shriver National Institute of Child Health and Human Development, Bethesda, MD 20892; 2 Eating and Addiction Section, Diabetes, Endocrinology, and Obesity Branch, National Institute of Diabetes and Digestive and Kidney Diseases, Bethesda, MD 20892

**Keywords:** cognition, dopamine, ErbB4, locomotion, motivation

## Abstract

Natural genetic variants of Neuregulin1 (NRG1) and its cognate receptor ErbB4 are associated with a risk for schizophrenia. Whereas most studies on NRG1-ErbB4 signaling have focused on GABAergic interneurons, ErbB4 is also expressed by midbrain dopaminergic neurons where it modulates extracellular dopamine (DA) levels. Here, we report that extracellular steady-state levels of DA are reduced in the medial prefrontal cortex (mPFC; −65%), hippocampus (−53%) and nucleus accumbens (NAc; −35%), but are elevated in the dorsal striatum (+25%) of ErbB4 knock-out mice (ErbB4 KOs) relative to wild-type controls. This pattern of DA imbalance recapitulates the reported prefrontal cortical reduction and striatal increase of DA levels in schizophrenia patients. Next, we report on a battery of behavioral tasks used to evaluate locomotor, cognitive and motivational behaviors in ErbB4 KOs relative to controls. We found that ErbB4 KOs are hyperactive in a novel open field but not in their familiar home cage, are more sensitive to amphetamine, perform poorly in the T-maze and novel object recognition (NOR) tasks, exhibit reduced spatial learning and memory on the Barnes maze, and perform markedly worse in conditioned place preference (CPP) tasks when associating cued-reward palatable food with location. However, we found that the poor performance of ErbB4 KOs in CPP are likely due to deficits in spatial memory, instead of reward seeking, as ErbB4 KOs are more motivated to work for palatable food rewards. Our findings indicate that ErbB4 signaling affects tonic DA levels and modulates a wide array of behavioral deficits relevant to psychiatric disorders, including schizophrenia.

## Significance Statement

Neuregulins (NRGs) and their major neuronal receptor in the brain, ErbB4, have been genetically associated with schizophrenia. In rodents, ErbB4 signaling has been shown to acutely regulate intrinsic interneuron excitability, synaptic plasticity, neuronal network activity and extracellular dopamine (DA) levels. Here we report that ErbB4 null mutant mice exhibit an imbalance of extracellular DA levels relative to controls, with increased levels in the dorsal striatum but reduced levels in the prefrontal cortex (PFC), hippocampus, and nucleus accumbens (NAc); a similar striatal and cortical DA imbalance has been reported in subjects with schizophrenia. Additionally, we show that ErbB4 null mice exhibit deficits in cognitive-related tasks, locomotor activity and motivation in a battery of behavioral assays previously reported to be associated with DA levels.

## Introduction

Neuregulins (NRGs) are a family of neurodevelopmental factors comprised of four genes (*NRG1–NRG4*) encoding proteins harboring a conserved epithelial growth factor-like (EGF-L) domain necessary for binding and activation of ErbB receptor tyrosine kinases (Buonanno, 2010; Mei and Nave, 2014). The major neuronal NRG receptor in the brain is ErbB4, where it regulates GABAergic neuronal migration, excitatory glutamatergic, cholinergic and inhibitory synapses onto GABAergic neurons (Chang and Fischbach, 2006; Zhong et al., 2008; Mitchell et al., 2013; Vullhorst et al., 2015), intrinsic interneuron excitability (Li et al., 2011; Janssen et al., 2012), synaptic plasticity (Kwon et al., 2005; Chen et al., 2010), neuronal network activity (i.e., γ oscillations; Fisahn et al., 2009; Andersson et al., 2012; Kawata et al., 2017), closure of the visual critical period (Sun et al., 2016; Grieco et al., 2019), and extracellular dopamine (DA) levels (Kwon et al., 2008; Namba et al., 2016; Skirzewski et al., 2018).

Importantly, numerous independent studies have identified a genetic association of natural variants of NRG1, NRG3, and ErbB4 with schizophrenia (Law et al., 2007; Kao et al., 2010; Greenwood et al., 2012; Joshi et al., 2014; Mostaid et al., 2016), as well as with altered electrophysiological properties of inducible pluripotential stem cells isolated from schizophrenia patients (Brennand et al., 2011). Postmortem analyses of the dorsal lateral prefrontal cortex (PFC) of patients indicate an alteration in the relative ratio of NRG1 and ErbB4 splice variants relative to controls (Law et al., 2006; Bertram et al., 2007; Chong et al., 2008). In this regard it is important that mice with targeted mutations in either *nrg1*, *nrg2*, *nrg3*, or *erbb4* exhibit a number of behavioral deficits that are relevant to traits affected in schizophrenia (Chen et al., 2010; Wen et al., 2010; Shamir et al., 2012; Lu et al., 2014; Hayes et al., 2016; Yan et al., 2018), and in two of the studies that tested the effects of antipsychotics in mutant mice, the behavioral deficits observed were improved (Tan et al., 2018; Yan et al., 2018).

In the cortex and hippocampus, cellular ErbB4 expression is confined to GABAergic interneurons (Vullhorst et al., 2009; Fazzari et al., 2010; Neddens and Buonanno, 2010; Neddens et al., 2011; Del Pino et al., 2013; Bean et al., 2014). ErbB4 levels are especially high in parvalbumin (PV)-positive interneurons, where receptor expression regulates γ oscillations (Fisahn et al., 2009; Chen et al., 2010; Fazzari et al., 2010; Wen et al., 2010; Shamir et al., 2012; Sun et al., 2016; Tan et al., 2018), a type of neuronal network activity important for working memory and other cognitive processes (Uhlhaas and Singer, 2010; Lewis et al., 2011; Miller et al., 2018). In contrast to the extensively studied function of ErbB4 in GABAergic interneurons, much less is known about the contribution of ErbB4 in mesencephalic DA neurons. Acute local administration of NRG1 (1 nm) by reverse microdialysis rapidly increases extracellular DA levels in the dorsal hippocampus, medial PFC (mPFC), and dorsal striatum within minutes (Kwon et al., 2008; Skirzewski et al., 2018). The increases of extracellular DA levels by NRG result from the activation of ErbB4 and downstream inhibition of the DA transporter (DAT), which is expressed on axonal processes (Skirzewski et al., 2018). Moreover, chronic disruption of NRG or ErbB4 signaling in knock-out (KO) mice alter tonic DA levels in the mPFC, hippocampus, and striatum (Kato et al., 2010, 2011; Mizuno et al., 2013; Golani et al., 2014; Tadmor et al., 2017, 2018; Skirzewski et al., 2018; Yan et al., 2018).

Despite the emerging literature associating NRG-ErbB signaling with DA function, how mutation of ErbB4 affects the nigrostriatal, mesocortical, and mesolimbic DA systems is presently unknown. Here we report that tonic extracellular levels of DA and its metabolites 3,4-dihydroxyphenylacetic acid (DOPAC) and homovanilic acid (HVA) in ErbB4 KOs are inversely disrupted between the dorsal striatum versus the mPFC, dorsal hippocampus, and nucleus accumbens (NAc) relative to controls. We also show that ErbB4 KO mice reproduce several behavioral deficits associated with altered striatal, hippocampal and/or cortical function that are relevant to psychiatric disorders, including schizophrenia.

## Materials and Methods

### Animals

We used a line of null ErbB4 KO mice originally developed by Tidcombe et al. (2003), which circumvents embryonic lethality by selective transgenic expression of ErbB4 in the heart driven by the myosin heavy chain promoter. Adult male ErbB4 KO and wild-type C57BL/6J controls (hereafter Ctrl) mice (two to five months old) were used. Mice were in a “clean” C57BL/6J background (>20 generations backcrossed to C57BL/6J) and housed in a conventional 12/12 h light/dark schedule with access to food and water *ad libitum* at the National Institutes of Health (NIH). Animal procedures were reviewed and approved by the NIH Animal Care and Use Committee.

### Reagents

Regular chow food (5001 Rodent Diet, 3.0 kcal/g with 29% energy derived from protein, 13% from fat, and 56% from carbohydrate) was obtained from LabDiet. Dustless Precision Pellets (14 mg, 3.6 kcal/g) were from BioServ. Amphetamine hydrobromide, neurochemical standards and HPLC reagents were from Sigma-Aldrich.

### Microdialysis

Extracellular DA, DOPAC, and HVA levels in the dorsal striatum, NAc, dorsal hippocampus, and mPFC were measured using *in vivo* microdialysis in freely moving mice, as previously reported (Skirzewski et al., 2018). Guide cannulas (stainless-steel 7 mm long, 21 gauge) were unilaterally implanted in the striatum (AP: +0.5 mm, L: 1.8 mm, V: 1.1 mm), NAc (AP: +1.4 mm, L: 0.5 mm, V: 2.2 mm), or mPFC (AP: +2.0 mm, L: 0.3 mm, V: 0.3 mm), and they were bilaterally implanted in the dorsal hippocampus (AP: −2.5 mm, L: 2.5 mm, V: 0.0 mm); the AP, L, and V values correspond to measurements relative to bregma, midsagittal sinus, and brain surface (Paxinos and Franklin, 2001). After surgery performed with 2% isoflurane/oxygen anesthesia, mice were allowed to recuperate for a week before starting microdialysis. Microdialysis probes were prepared in the laboratory, as described (Hernandez et al., 1986), and consisted of an 8-mm-long, 26-gauge, stainless steel tube plus a 2-mm-long (mPFC, striatum) or 1-mm-long (NAc, hippocampus) cellulose hollow fiber tip (18 kDA MWCO, SpectrumLabs Inc) that protruded from the guide cannula. Modified ACS fluid (136 mm NaCl, 3.7 mm KCl, 2.2 mm CaCl_2_, 1 mm MgCl_2_, and 10 mm NaHCO_3_ at pH 7.4) was perfused through the probes at a flow rate of 1 μl/min.

**Figure 1.**
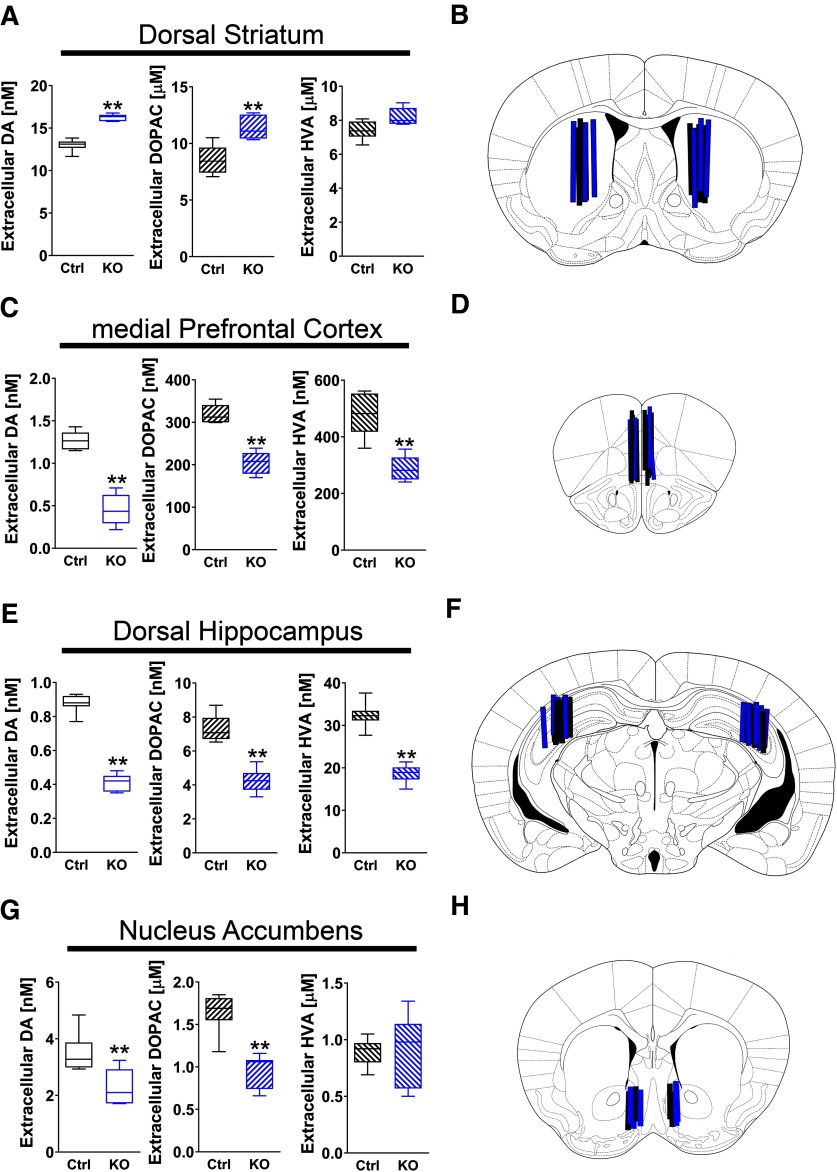
Tonic extracellular levels of DA and its metabolites are altered in ErbB4 KO mice relative to WT controls. Tonic extracellular levels of DA, DOPAC, and HVA (panels on left), and unilateral or bilateral location of probes used for microdialysis (schematic depiction on right), are shown for the (***A***, ***B***) dorsal striatum, (***C***, ***D***) medial prefrontal cortex, (***E***, ***F***) dorsal hippocampus, and (***G***, ***H***) NAc of ErbB4 KO (KO, blue; *n* = 6–7) and control (Ctrl, black; *n* = 6–7) mice. Data are expressed as the mean ± SEM; **p < 0.01.

Five samples were collected consecutively for 15 min from each mouse into tubes containing 5 μl of 100 mm HCl + 1 mm EDTA, which were immediately frozen in dry-ice following collection to prevent catecholamine oxidation. Samples from two genotype-matched mice were pooled to enable measurement of DA collected from the hippocampus and mPFC using electrochemistry (see HPLC-electrochemical detection). To estimate extracellular concentration of DA, DOPAC, and HVA, the percentage of recovery was obtained *in vitro* for each microdialysis probe. Mice used to measure DA levels were sacrificed following sample collection to confirm the anatomic placement of the probe. Brains were fixed by trans-cardiac perfusion using 30 ml PBS, pH 7.4, followed by 30-ml 4% formaldehyde in PBS, sectioned (50 μm thick), and Nissl stained to verify probe placement (Fig. 1*B*,*D*,*F*,*H*). Brains with misplaced microdialysis probes were excluded from data analysis.

### HPLC-electrochemical detection

The extracellular content of DA, DOPAC, and HVA from dialyzed samples was analyzed by injecting the total sample volume into an isocratic HPLC system with electrochemical detection (Model LC-4C, BASi), essentially as published (Skirzewski et al., 2018; Yan et al., 2018). The order and elution time of the neurochemicals was as follows: DOPAC ∼7.0 min, DA ∼9.5 min, and HVA ∼21.9 min. DA, DOPAC, and HVA were measured by matching the area under the curve (AUC) for each sample versus the AUC for the curves for each standard; then the amount was corrected for the percent recovery for the dialysis probe corresponding to each molecule. Detection sensitivity limit was set to 1 nA for DA, DOPAC, and HVA, estimated to correspond to 2.4, 1.3, and 2.7 pg, respectively.

### Behavioral testing battery

We performed and compared a battery of behavioral tasks in ErbB4 KOs and Ctrl mice (three to five months old) using cohorts that varied in number (5–13 mice) depending on the test used. As previously suggested (Crawley, 2007), and to minimize/optimize the number of animals used in this study, we used eight independent cohorts of mice that were tested in no more than five consecutive behavioral tasks performed in the following order. Cohorts 1 and 2: home-cage locomotor activity, open field test, novel object recognition (NOR), T-maze, and Barnes maze. Cohort 3: auditory Pavlovian conditioning, progressive ratio (PR), and amphetamine challenge test. Cohort 4: two-bottle sucrose preference, auditory Pavlovian conditioning, PR, and conditioned place preference (CPP) to palatable food. Cohort 5: two-bottle sucrose preference, PR, and CPP to palatable food. Cohort 6: amphetamine challenge test. Cohort 7: energy balance/food consumption. And cohort 8: body weight analysis. All behavioral tests were performed during the light period (12/12 h light/dark cycle).

#### Locomotor activity in home cage

ErbB4 KO and Ctrl mice were independently housed in a home cage (19 × 18 × 40 cm) with regular bedding, free access to food and water, and standard 12/12 h light/dark schedule for 96 h. Horizontal locomotor activity was continuously recorded by placing the home cage within an infrared beam-equipped frame (Columbus Instruments).

#### Open field test

Novelty induced locomotor activity was tested in a 35 × 35 × 35 cm open field arena. Mice were independently placed in one corner of the arena and horizontal movements recorded during 30 min with Any-Maze Behavior Video Tracking Software V 4.72 (Stoelting Co). The open field chamber was cleaned between sessions with 70% v/v alcohol solution.

#### Amphetamine challenge

Systemic administration of amphetamine (intraperitoneal) in rodents induces an overall increase in locomotor activity (Bardo et al., 1990), and altered basal extracellular DA levels in the brain affects their amphetamine-induced locomotor response (Yan et al., 2018). ErbB4 KO and Ctrl mice were challenged with different doses of amphetamine (0.0, 0.5, 1.5, 2.5, and 3.5 mg/kg, i.p.). As described for the open field test (see above), horizontal locomotor activity was initially recorded in an open field arena (35 × 35 × 35 cm) for 60 min before administering a single injection of amphetamine or vehicle (0.9% NaCl). Locomotor activity was then recorded for an additional 60 min. Each mouse was used once in this challenge regardless of treatment (amphetamine or vehicle).

#### NOR

Memory function in ErbB4 KO and Ctrl mice was tested using the NOR task with minimized spatial and contextual factors, as previously described (Forwood et al., 2005). Briefly, NOR used a Y-shaped Plexiglas apparatus containing three arms (120° angled) 40-cm-long, 9-cm-wide, and 13-cm-high walls each. The NOR task was assessed with two consecutive 5-min trials (sample and choice) separated by a 2-min intertrial interval (ITI). The sample trial consisted of setting up two arms with identical objects placed at the distal portion of each arm. The remaining arm (start arm) did not have any object and was equipped with a guillotine door (30 cm away from the center of apparatus) to prevent the mouse’s free exploration. The sample trial started by raising the guillotine door at the start arm to allow the mouse to freely explore the apparatus and objects. During the choice trial, one of the objects was replaced by a new object of different shape and texture, but of similar size. The time mice spent in arms with novel versus familiar objects were video recorded using Any-Maze (Stoelting), and the exploration ratio (novel vs familiar) was assessed. The apparatus and objects were wiped down with 70% v/v ethanol between sessions.

#### T-maze

Spontaneous alternation behavior in mice reflects a sufficient level of reference and working memory to successfully explore for novel environments (Deacon and Rawlins, 2006). As previously described (Skirzewski et al., 2018), the test was assessed in an opaque plastic enclosed T maze apparatus consisting of one start arm where the mouse started the trial and two choice arms equipped with Plexiglas doors at the entrance. Briefly, the task session consisted of a “sample” and a “choice” trial separated by 15-s ITI where the mouse remained in a clean home cage. The sample trial started after gently placing the mouse at the end of the start arm and allowing it to freely explore either the left or the right arm. The arm was defined as “chosen” when the mouse placed the four paws and tail inside an arm. Once inside the arm, the mouse was restricted to this compartment for 30 s. The mouse was then gently retrieved out of the apparatus for ITI. Lastly, with all the doors opened, the choice trial was initiated by placing the mouse at the start arm and allowing it to freely explore the apparatus. Whether the mouse chose to explore the unvisited arm (alternation) or the previously visited arm (no alternation) was recorded. Spontaneous performance was scored in each mouse over three sessions that were separated by 2 h each.

#### Barnes maze

The Barnes maze was used to assess spatial learning memory, as previously described (Sunyer et al., 2007; Skirzewski et al., 2018). The apparatus consisted of an elevated white Plexiglas circular table (92 cm in diameter) with 20 equally spaced holes (5 cm in diameter, 7.5 cm between holes) along the perimeter. One of the holes (target) contained a hidden box underneath the table that allowed mice to hide from two mild stressors (85-db background noise/900 lux light) applied during the training trials (see below). The remaining 19 holes did not offer escape to the mild stressors and were sequentially numbered +1 to +9, opposite to target, and −9 to −1. Holes +1 and −1 corresponded to the adjacent holes to target. The task consisted in two phases: four training sessions (one per day, days 1–4) and probe trial (day 5). The training sessions consisted in 4 trials/session during four consecutive days (30-min ITI). Each trial was 3 min long or until the mouse escaped to the hidden box underneath the target, whichever came first. Time mice spent finding the target (latency time) and number of errors (nose pokes in other holes) for each trial were scored from day 1 to 4. Probe trial (day 5) was recorded once per mouse during 90 s, and the hidden box was removed from the apparatus. Latency time mouse entered for the first time to the correct quadrant (1/4 of the platform area including target and holes ±1 and ±2), total time spent in correct quadrant, and number of nose pokes to target and other holes were recorded using Any-Maze (Stoelting). The apparatus was wiped between trials with a 70% v/v alcohol solution.

#### Two-bottle sucrose test

The two-bottle sucrose test paradigm was used to determine anhedonia in ErbB4 KOs and Ctrl mice as previously described (Hutchison et al., 2018). Mice housed individually were habituated to drink tap water from two identical bottles during 24 h. Then, mice were given a free choice to drink either 2% (v/v) sucrose solution or tap water in two bottles and intake scored by weighting the bottles every 24 h for four consecutive days. To avoid side preference, the location of sucrose and water bottles were alternated every 24 h. The sucrose preference ratio was calculated as the amount of sucrose solution consumed relative to the total amount of liquid consumed (sucrose solution intake/[sucrose solution intake + water intake]).

#### CPP

Using the five-test design (Hutchison et al., 2018), food-restricted mice (85% body weight) were tested for their ability to form conditioned spatial associations to palatable food (14-mg dustless precision pellets, BioServ) in the CPP paradigm. The CPP apparatus consisted of two large compartments (20 × 20 × 20 cm) with different visual and tactile cues, which were separated by a smaller center compartment (10 × 10 × 20 cm). First, mice underwent in a pre-training session to identify their natural place preference by allowing them to freely explore the apparatus during 20 min. A biased design at days 1, 3, 5, 7, and 9 was used to administer a neutral stimulus (no food) in the naturally preferred side (PS) or 20 palatable food pellets sparsely distributed on the floor of the least PS (LPS). Mouse exploration was restricted to either PS or LPS during 15 min depending of stimulus (neutral or palatable food pellets). Probe sessions were held at days 2, 4, 6, 8, and 10 and consisted in a single trial to let mice to freely explore the entire apparatus during 20 min with no palatable food provided. Time spent at each compartment was recorded (LPS vs PS) using Any-Maze (Stoelting), and preference ratio estimated as the time spent in the LPS divided by the total time spent in PS and LPS.

#### Auditory Pavlovian conditioning

Food restricted (85% body weight) ErbB4 KO and Ctrl mice were used to assess deficits in associative learning behavior using a previously described paradigm (Berridge and Robinson, 2003). Experiments were performed in a sound-attenuated operant chamber equipped with a fan to provide white noise and ventilation during the experiment (20 × 20 × 20 cm, ENV-300; Med Associates). Briefly, mice were trained during 30 min to retrieve a palatable food reward (14-mg dustless precision pellets, BioServ) according to the following trial schedule: 2-s tone (85 db) → 1-s delay → 5-s-long light on inside reward magazine → mouse retrieve reward within when reward magazine is illuminated → 15-s ITI → repeat trial. If the mouse nose poked the reward magazine at any other time different from when the reward was active (light on), a time out was applied (5-s-long house light on) and the schedule resumed with 15-s ITI. Daily single sessions were performed for eight consecutive days and percentage of accuracy (rewards retrieved vs total trials) and premature responses (time out) scored.

#### PR

This task was used to test the motivation and willingness of ErbB4 KO and Ctrl mice to work for palatable food rewards (Berridge and Robinson, 2003). Mice were kept at 85% free-feeding body weight and conditioning was performed in sound-attenuated operant chambers (ENV-300; Med Associates). Briefly, chambers were equipped with two illuminated nose-poke apertures on both sides of a reward magazine that dispensed palatable food pellets (14-mg dustless precision pellets, BioServ), and a fan that provided white noise and ventilation to the chamber during the session (Fig. 5*A*). One nose-poke aperture was set as active and the other as inactive, with the active location counterbalanced across mice. Nose poking in the active aperture resulted in the delivery of one palatable food pellet, while responses in the inactive aperture were recorded but had no consequence. Before conditioning, mice were habituated to retrieve rewards delivered at variable interval schedules (range 5–100 s) for 30 min and nose poke responses had no consequence. Then, mice were trained in a fixed ratio 1 (FR1) schedule of reinforcement where each nose poke in the active aperture resulted in the delivery of one palatable food pellet. Our criteria for stable performance was the collection of 30 food pellets in a 30-min-long session during three consecutive days. Once criteria were met, mice were progressively transferred to FR3 (three responses in active aperture – one reward) and FR5 (five responses in active aperture – one reward) schedules following the same criteria performance. Finally, one PR7 schedule session was assessed daily during five consecutive days to test for the mouse’s motivation to work for rewards. PR7 session consisted in a progressive increment of the required nose pokes into the active aperture (7, 14, 21, 28, 35, etc.) per trial to obtain one palatable food pellet. The total number of palatable pellets collected (breaking point) and number of nose pokes in the active/inactive holes in a 2-h-long session or after 60 min of inactivity (whichever occurred first) were recorded.

#### Food intake, body weight, body composition, and energy balance

Three-month-old ErbB4 KO and Ctrl mice were single housed under standard conditions (12/12 h light/dark cycle, 21–22°C) with *ad libitum* access to water and regular chow diet (LabDiet, 5001). Following one week of habituation, chow was presented in Rodent Cafes (OYC Americas), and food intake and body weights were recorded daily for 10 d. Food intake was measured by manually weighing the Rodent Cafes every 24 h and converting the weight consumed into calories using the metabolizable energy content. Body composition (fat and lean mass) was measured on the first and tenth day by 1H NMR spectroscopy (EchoMRI-100H; Echo Medical Systems LTD). Energy expenditure was calculated using the following equation: energy expenditure = food intake − (Δ fat mass + Δ lean mass), as previously reported (Guo and Hall, 2009; Ravussin et al., 2013). Food consumption was presented as the total of energy intake (kcal) during 20 min and normalized by the mouse body weight before the session.

### Statistics

Data were analyzed by using GraphPad Prism v8.2.1. Relative levels of DA and metabolites were estimated by comparing the AUC of each analyte with respect to the AUC of their corresponding standards. Additionally, extracellular concentration was corrected relative to the percentage of recovery for each microdialysis probe, which was calculated to be between 6–8% for a 1-mm-long tip and 10–15% for a 2-mm-long tip. All data were examined for normal distribution with D’Agostino and Pearson normality test. Unpaired two-tailed Student’s *t* test or two-way ANOVA for repeated measures and Sidak’s multiple comparisons *post hoc* analysis was used with data showing normal distribution. If data set did not show normal distribution, two-tailed Mann–Whitney *U* test was used. Data are represented as the mean ± SEM, and significance was set at *p* < 0.05.

## Results

### ErbB4 KO mice show altered extracellular DA levels and metabolites across four different brain regions

Although pharmacological or genetic manipulations of the NRG/ErbB4 signaling pathway have been reported to affect DA levels (Kato et al., 2010, 2011; Mizuno et al., 2013; Golani et al., 2014; Skirzewski et al., 2018; Yan et al., 2018), a systematic study of how genetic ablation of ErbB4 in adult mice alters tonic extracellular DA levels in distinct brain regions has not been undertaken. To address this point, we performed in vivo microdialysis coupled to HPLC/electrochemical neurochemical detection in freely moving ErbB4 KO and Ctrl mice to systematically measure extracellular DA, DOPAC, and HVA levels. Samples were collected from major dopaminergic brain regions comprised by the nigrostriatal (dorsal striatum), mesocortical (mPFC and dorsal hippocampus), and mesolimbic (NAc) systems. We found that extracellular DA levels (ErbB4 KO: 16.26 ± 0.16 nm vs Ctrl: 13.00 ± 0.29 nm; *U* = 0, *p* = 0.0022) and DOPAC levels (ErbB4 KO: 11.36 ± 0.41 μm vs Ctrl: 8.55 ± 0.52 μm; *U* = 2, *p* = 0.0087) were elevated in the dorsal striatum of ErbB4 KOs (*n* = 6) relative to controls (*n* = 6), whereas HVA levels (ErbB4 KO: 8.20 ± 0.21 μm vs Ctrl: 7.41 ± 0.22 μm; *p* > 0.05) were unchanged (Fig. 1*A*). The observation that DOPAC levels were higher, whereas HVA levels were similar between groups, is consistent with the notion that DA clearance in the striatum is mostly mediated by DAT uptake.

In contrast to the dorsal striatum, we found that extracellular levels of DA and its metabolites DOPAC and HVA were lower in both the mPFC (DA ErbB4 KO: 0.45 ± 0.08 nm, *n* = 6 vs Ctrl: 1.27 ± 0.05 nm, *n* = 6, *U* = 0, *p* = 0.0022; DOPAC ErbB4 KO: 205.10 ± 10.91 nm vs Ctrl: 319.10 ± 8.92 nm, *U* = 0, *p* = 0.0022; HVA ErbB4 KO: 288.40 ± 17.50 nm vs Ctrl: 478.90 ± 30.94 nm; *U* = 0, *p* = 0.0022; Fig. 1*C*) and dorsal hippocampus (DA ErbB4 KO: 0.41 ± 0.02 nm, *n* = 6 vs Ctrl 0.88 ± 0.02 nm, *n* = 7, *U* = 0, *p* = 0.0012; DOPAC ErbB4 KO: 4.25 ± 0.28 nm vs Ctrl 7.38 ± 0.76 nm, *U* = 0, *p* = 0.0012; HVA ErbB4 KO: 18.66 ± 0.87 nm vs Ctrl 32.31 ± 1.13 nm, *U* = 0, *p* = 0.0012; Fig. 1*E*) of ErbB4 KOs. Of note, we observed a similar imbalance of extracellular DA levels between striatal and extra-striatal structures in mice lacking the ErbB4 ligand NRG2 (Yan et al., 2018), suggesting that alterations in NRG-ErbB4 signaling in the whole brain differentially affect the nigrostriatal and mesocortical systems.

Because tonic DA levels are differentially altered in ErbB4 KO nigrostriatal (Fig. 1*A*) and mesocortical structures (Fig. 1*C*,*E*) relative to Ctrl mice, and the mesolimbic NAc comprises an anatomic structure that also receives inputs from the VTA, we were interested in investigating how mutation of ErbB4 affects tonic DA within the NAc. Interestingly, we found that extracellular DA levels (ErbB4 KO: 2.28 ± 0.22 nm, *n* = 7 vs Ctrl: 3.51 ± 0.25 nm, *n* = 7; *U* = 2, *p* = 0.0023) and DOPAC levels (ErbB4 KO: 0.94 ± 0.07 μm vs Ctrl 1.64 ± 0.09 μM; *U* = 0, *p* = 0.0006), but not HVA (ErbB4 KO 0.88 ± 0.12 μM; Ctrl 0.89 ± 0.05 μM, *p* > 0.05), were significantly reduced in ErbB4 KOs relative to Ctrl (Fig. 1*G*). The effects of ErbB4 ablation on NAc DA and DOPAC, but not on HVA, are consistent with relatively high expression levels of DAT in the NAc (ventral striatum) used to clear extracellular DA.

### ErbB4 KO mice are hyperactive and hypersensitive to amphetamine

**Figure 2.**
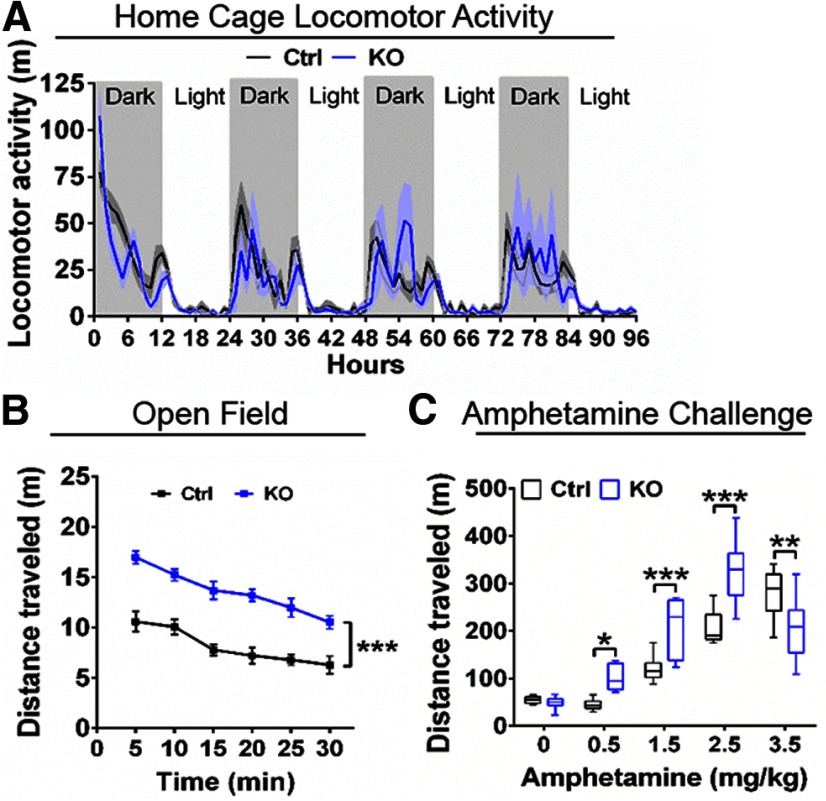
ErbB4 KO mice are hyperactive in novel environments and sensitive to systemic amphetamine. ***A***, Horizontal locomotor activity of control (Ctrl, black) and ErbB4 KO (KO, blue) mice scored in their home cages for 96 h (*n* = 8/genotype, *p* > 0.05). ***B***, Locomotor activity of ErbB4 KO mice (KO, blue) is significantly elevated relative to controls (Ctrl, black) in the novelty open field test (*n* = 9/genotype, *F*_(2,24)_ = 36.77, *p* < 0.0001). ***C***, Locomotor activity of controls (Ctrl, black) and ErbB4 KO mice (KO, blue) following administration (intraperitoneal) of 0–3.5 mg/kg amphetamine. ErbB4 KO mice (0 mg/kg, *n* = 9; 0.5–3.5 mg/kg, *n* = 8) show locomotor hypersensitivity to systemic amphetamine administration at different doses as in contrast to Ctrl mice (0 mg/kg, *n* = 8; 0.5 mg/kg, *n* = 10; 1.5 and 2.5 mg/kg, *n* = 8; 3.5 mg/kg, *n* = 9; *F*_(1,74)_ = 16.85, *p* = 0.0001); *p < 0.05, **p < 0.01, ***p < 0.001.

Changes in extracellular levels of neuromodulators in the striatum and neocortex have been associated with altered behaviors in rodents. For example, elevated tonic extracellular DA levels in the striatum have been associated with increased locomotor activity in rodents (Di Chiara and Imperato, 1988; Bardo et al., 1990). For this reason, we analyzed horizontal locomotor activity of ErbB4 KO mice relative to Ctrl in their home cage (i.e., habituated) and in the open field (i.e., novelty). Interestingly, we found that activity of ErbB4 KOs and Ctrl did not differ when recorded in their home cages during a 96-h period (*n* = 8/genotype, *p* > 0.05; Fig. 2*A*), but in the open field ErbB4 KO mice traveled longer distances than Ctrl mice (ErbB4 KO: 81.6 ± 3.0 m, *n* = 9; control: 48.7 ± 2.7 m, *n* = 9; *F*_(2,24)_ = 36.77 *p* < 0.0001; Fig. 2*B*). Moreover, ErbB4 KOs were hypersensitive to systemic amphetamine (intraperitoneal) relative to Ctrl (*F*_(1,74)_ = 16.85, *p* = 0.0001; Table 1; Fig. 2*C*), as their total horizontal locomotor activity was higher at doses between 0.5–2.5 mg/kg and showed a reverse U-shaped response when the dose reached 3.5 mg/kg (intraperitoneal). The reversed U-shaped curve in response to amphetamine is consistent with previous work showing that that high doses of amphetamine resulted in stereotypical behaviors, rather than elevated horizontal locomotor activity (Segal, 1975). Moreover, these findings are also consistent with prior studies reporting ErbB4 KO mice are hyperactive when exposed to novel environments and to augmented basal levels of striatal DA in KOs (Shamir et al., 2012; Marchisella et al., 2018).

**Table 1:** Total horizontal locomotor activity (meters) recorded for 60 min from ErbB4 KO and Ctrl mice that received systemic injections (intraperitoneally) of d-amphetamine (mg/kg)

mg/kg	Ctrl	*n*	ErbB4 KO	*n*	*p*
0.0	54.6 ± 2.5	8	49.3 ± 4.1	9	n.s.
0.5	44.5 ± 3.3	10	105.8 ± 9.5	8	<0.05
1.5	121.2 ± 9.4	8	207.1 ± 21.8	8	<0.005
2.5	206.2 ± 12.4	8	326.6 ± 22.8	8	<0.001
3.5	279.1 ± 17.1	9	207.0 ± 23.3	8	<0.01

n.s., no significant differences.

### ErbB4 KO mice exhibit deficits in spatial learning memory

**Figure 3.**
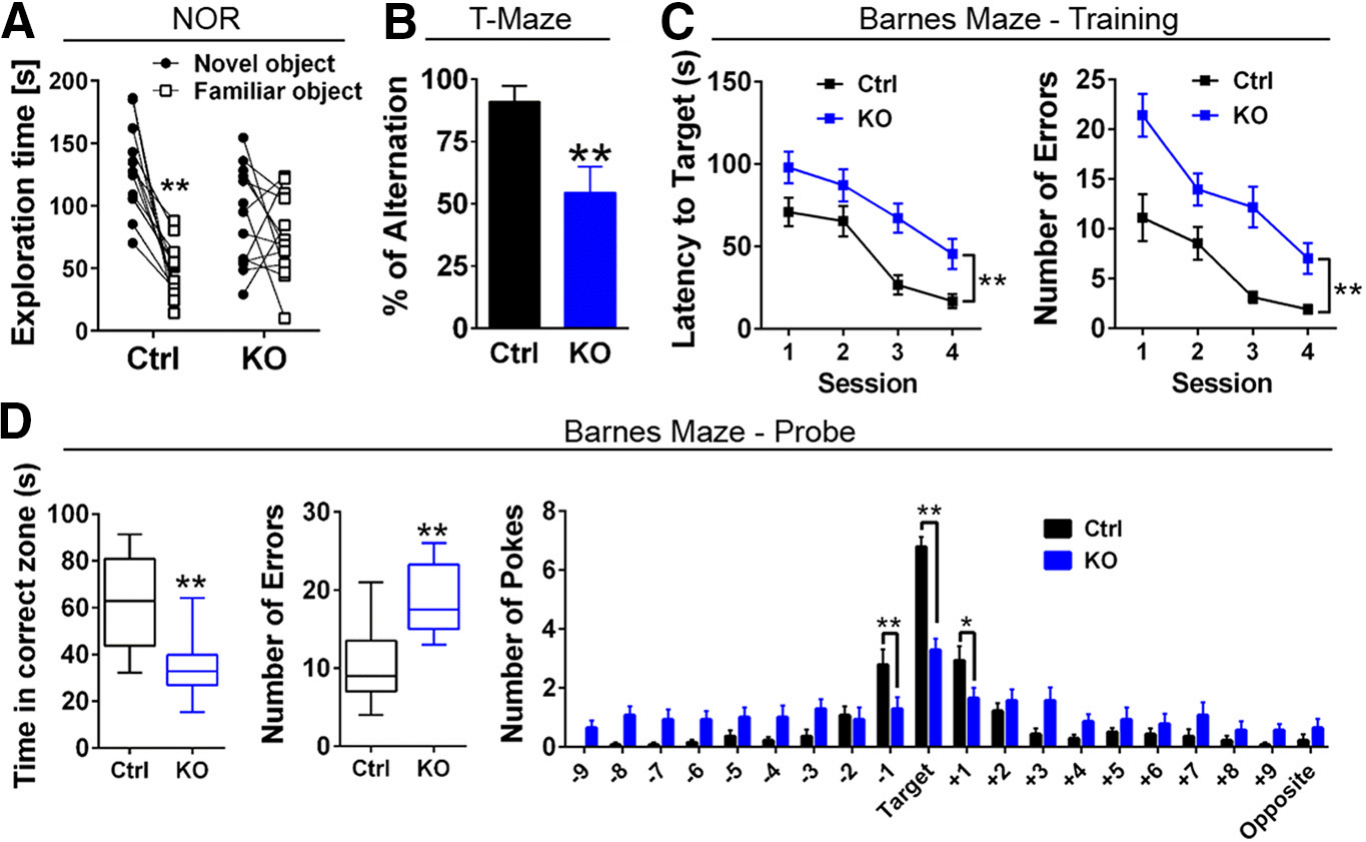
ErbB4 KO mice exhibit spatial learning and memory deficits. ***A***, ErbB4 KO mice (*n* = 13) show deficits during the NOR test as compared with control mice (Ctrl, *n* = 13). ErbB4 KO mice were impaired to differentiate between the novel versus familiar object, assessed by the total time each object was explored during the probe session. ***B***, In contrast to control mice (Ctrl, black, *n* = 11), ErbB4 KO mice (KO, blue, *n* = 11) had lower spontaneous alternation in the T-maze. ***C***, Barnes maze (*n* = 14/genotype) revealed learning deficits in ErbB4 KO mice to locate the target during the training sessions (days 1–4), including higher latency time to find the target (left panel) and more errors (right panel). ***D***, Consistently, the probe session (day 5) indicated that ErbB4 KO mice were impaired to locate the target, as the time spent in the correct zone was reduced (left panel), the number of incorrect nose pokes (errors) were higher (middle panel), and the number of nose pokes in the target and holes +1 and −1 were significantly lower than controls (right panel); *p < 0.05, **p < 0.01.

Numerous studies in humans (Egan et al., 2001; Cools and D'Esposito, 2011), non-human primates (Arnsten et al., 1994; Williams and Goldman-Rakic, 1995), and rodents (Spellman et al., 2015; Cassidy et al., 2016) have shown the importance of an “inverted U-shape” relationship between optimal DA levels in PFC and performance on cognitive-related tasks. Based on this relationship, we reasoned that performance of ErbB4 KOs could be affected in hippocampal-dependent and PFC-dependent tasks. We therefore compared ErbB4 KOs and Ctrl in a battery of behavioral tasks to evaluate learning, spatial, and working memory reliant on these regions. ErbB4 KOs (*n* = 12) underscored relative to Ctrl (*n* = 12) in NOR (Fig. 3*A*), as ErbB4 KOs randomly explored both novel and familiar objects during the probe session (novel 91 ± 11 s vs familiar 75 ± 9 s; *p* > 0.05), whereas Ctrl mice spent significantly more time with novel objects (novel 129 ± 10 s vs familiar 46 ± 6 s; *t*_(12)_ = 6.47, *p* < 0.0001). Additionally, ErbB4 KO mice failed to spontaneously alternate to explore both arms between consecutive trials in the T-maze (54.6 ± 10.3%, *n* = 11), in contrast to Ctrl mice (Ctrl: 90.9 ± 6.5%, *n* = 11), indicating deficits in working memory (*U* = 21.5, *p* = 0.0055; Fig. 3*B*). Finally, assessing spatial learning using the Barnes maze, we found that ErbB4 KOs (*n* = 14) are impaired in learning the location of the target hole relative to Ctrl, as manifested by the increased latency times (*F*_(1,26)_ = 11.56, *p* = 0.0022) and errors (*F*_(1,26)_ = 20.13, *p* = 0.0001) per session as compared with Ctrl (*n* = 14; Fig. 3*C*). Moreover, ErbB4 KOs showed deficits in spatial memory during the Barnes maze probe session (Fig. 3*D*), as suggested by the reduced time mice spent in the correct zone (ErbB4 KO 35 ± 13 s vs Ctrl 63 ± 5 s; *t*_(26)_ = 4.478, *t*_(26)_ = 4.478, *p* < 0.0001), the higher number of nose pokes errors (ErbB4 KO 19 ± 1 vs Ctrl 11 ± 1; *t*_(26)_ = 4.528, *p* < 0.0001), and their overall lower correct/incorrect hole nose-pokes score (*F*_(1,520)_ = 4.663, *p* = 0.0313). These findings suggest that the NRG-ErbB4 signaling pathway plays a relevant role in memory consolidation.

### ErbB4 KO mice show increased willingness to work for palatable food rewards unrelated to energy requirement mechanisms

**Figure 4.**
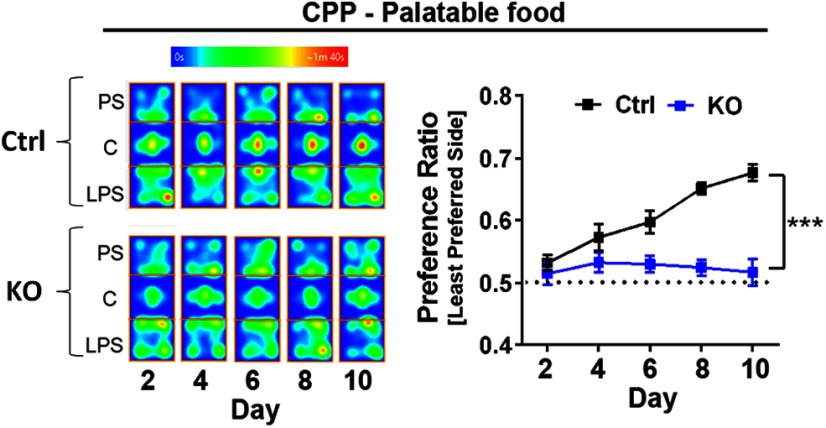
Cue-reward spatial association to palatable food is impaired in ErbB4 KO mice. To evaluate for spatial-cue associations to rewards, a five-test design (days 2, 4, 6, 8, and 10) was used to allow assessment of the development of a CPP to palatable food rewards. Food-restricted mice were conditioned to receive palatable food rewards (14-mg dustless pellets, BioServ) in a previously assigned LPS of the apparatus versus no food at the PS in two consecutive training sessions at days 1, 3, 5, 7, and 9. The left panel is a representative heat map of ErbB4 KO (KO) and control (Ctrl) mice exploring the PS, center (C), and LPS of the apparatus across test sessions and the right panel shows the preference ratio of mice to develop CPP. Control mice (*n* = 15) progressively conditioned across sessions to spent more time in the LPS, yet ErbB4 KO mice (*n* = 16) did not develop spatial-cue associations to palatable food rewards (two-way ANVOA genotype effect: *F*_(1,29)_ = 32.78, *p* < 0.0001, two-way ANVOA time effect: *F*_(4,116)_ = 8.619, *p* < 0.0001). Data represents the mean ± SEM; ***p < 0.005.

The mesolimbic DA pathway, which projects from VTA to the NAc, is critical for the signal incentive salience regulating reinforcement/reward-related motor function learning (Schultz et al., 1997; Mohebi et al., 2019). Importantly, alterations in this pathway have been implicated in many human disorders including Parkinson’s disease, schizophrenia and drug addiction (Salamone et al., 2007; Weinstein et al., 2017). Because we found NAc DA levels are reduced in ErbB4 KO mice (Fig. 1*G*), we assessed whether this altered pattern of DA was associated with behavioral changes in motivation and reinforcement learning. We initially tested anhedonia in mice using the two-bottle sucrose preference test (Nestler and Hyman, 2010). Consistent with prior observations (Shamir et al., 2012), we found that ErbB4 KOs did not show altered preference for a sucrose solution as the ratio of sucrose-water consumption was similar to that of Ctrl mice (KO: 0.83 ± 0.02, *n* = 11; vs Ctrl: 0.80 ± 0.01, *n* = 12; *p* = 0.0525). Next, we performed a CPP task (Fig. 4) to assess the development of reward-location associations. We found that, in stark contrast to the robust reward-associated place preference manifested by Ctrl mice (*n* = 15), ErbB4 KOs (*n* = 16) did not exhibit place preference association to palatable food pellets (two-way ANOVA genotype effect: *F*_(1,29)_ = 32.78, *p* < 0.0001, two-way ANOVA time effect: *F*_(4,116)_ = 8.619, *p* < 0.0001; Fig. 4). While these results could be interpreted as a failure of ErbB4 KOs to form cue-reward associations, previous reports have emphasized that CPP in rodents heavily relies on a preserved hippocampal system for spatial navigation (Ferbinteanu and McDonald, 2001; White et al., 2005; Ito et al., 2008). Because we observed that ErbB4 KOs exhibit deficits in spatial memory (Fig. 3*C*,*D*), we reasoned it was important to use other cue-reward behavioral paradigms before interpreting our CPP findings.

**Figure 5.**
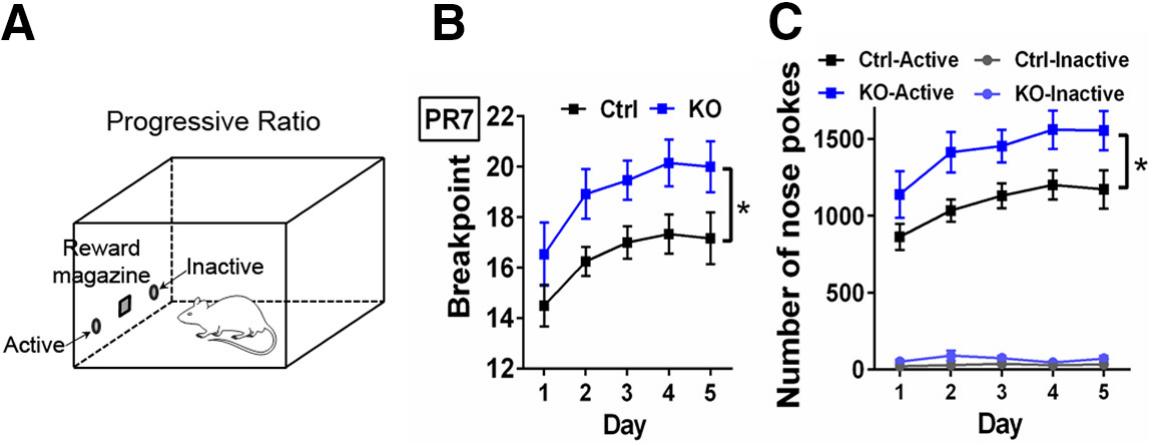
ErbB4 KO mice show increased motivation to work for palatable rewards. ***A***, Experimental set-up of a PR (PR7) schedule in food-restricted (∼85% free-food body weight) ErbB4 KO (KO, *n* = 13) and control (Ctrl, *n* = 12) mice. The PR7 is an instrumental learning paradigm where mice are trained to nose poke an active hole to retrieve a palatable reward (14-mg dustless precision pellet, BioServ) that increasingly becomes more difficult to obtain with each subsequent reward delivery (first pellet at seven nose pokes, second pellet at 14 nose pokes, etc.). ***B***, Interestingly, ErbB4 KO mice show increased motivation to obtain palatable food rewards than Ctrl mice as their break point (number of rewards collected in a total of 120 min) was consistently larger at each session (days 1–5; *F*_(1,23)_ = 5.565, *p* = 0.0272). ***C***, The larger number of rewards obtained by ErbB4 KO mice were attributed to goal-directed instrumental behaviors, and not to an overall increased locomotor activity because ErbB4 KO mice specifically and more frequently nose poked the active hole than Ctrl mice (*F*_(1,23)_ = 6.332, *p* = 0.0193). Data are expressed as the mean ± SEM; *p < 0.05.

As alternatives to CPP, we used an auditory classical conditioning Pavlovian task and PR paradigm to model cue-reward associations, instrumental learning and reward in ErbB4 KOs (Salamone and Correa, 2002; Berridge and Robinson, 2003). The classical conditioning Pavlovian task did not uncover deficits in associative learning behaviors between genotypes (two-way ANOVA: *F*_(1,13)_ = 1274, *p* = 0.2794), as ErbB4 KOs (*n* = 7) and Ctrls (*n* = 8) learned similarly to associate an auditory cue-tone with the delivery of a palatable food reward across consecutive daily training sessions (two-way ANOVA: *F*_(7,91)_ = 170.7, *p* < 0.0001). Next, to evaluate instrumental learning, we trained mice to nose-poke a hole to earn a palatable food reinforcer under FR schedules. We found that ErbB4 KOs (*n* = 13) reached criteria faster than Ctrls (*n* = 12) at multiple FR schedules: FR1 (ErbB4 KO 3.4 ± 0.1 d vs Ctrl 5.2 ± 0.2 d, *t*_(23)_ = 7.22, *p* < 0.0001), FR3 (ErbB4 KO 3.2 ± 0.2 d vs Ctrl 3.9 ± 0.3 d, *t*_(23)_ = 2.242, *p* = 0.0174), and FR5 (ErbB4 KO 3.0 ± 0.0 d vs Ctrl 3.7 ± 0.4 d, *t*_(23)_ = 1.849, *p* = 0.0387). Next, mice underwent a PR schedule to further evaluate their willingness to work for rewards (Fig. 5*A*). Unexpectedly, ErbB4 KOs outperformed Ctrl mice as defined by their higher break point average across five consecutive probe sessions (*F*_(1,23)_ = 5.565, *p* = 0.0272; Fig. 5*B*). Importantly, the performance of ErbB4 KO and Ctrl mice to retrieve rewards was goal directed, as the number of nose pokes to the active hole was significantly higher than to the inactive hole (*F*_(1,23)_ = 6.332, *p* = 0.0193; Fig. 5*C*). Taken together, these findings suggest that ErbB4 KOs do not show deficits in cue-reward associations and instrumental learning, as would be suggested by the CPP results alone (Fig. 4). Instead, ErbB4 KOs are more motivated to work for palatable food rewards than their Ctrl littermates (Fig. 5).

**Figure 6.**
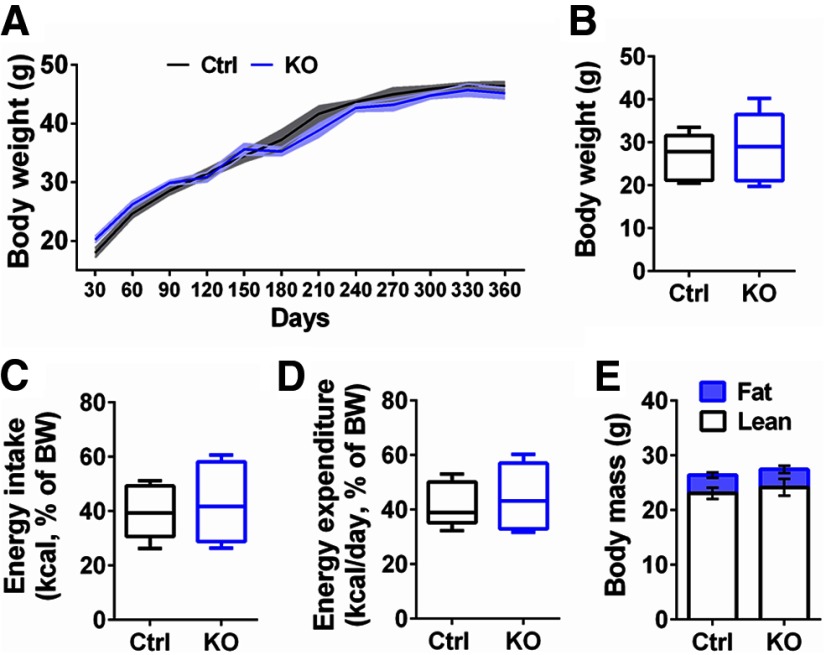
ErbB4 KO mice do not show differences in energy balance and body composition. ***A***, ErbB4 KO mice (KO, blue) kept with regular chow and water *ad libitum* in their home cages did not show differences with control (Ctrl, black) mice in body weight when continuously monitored every 30 d up to 360 d (*n* = 7/genotype, *p* > 0.05). The shaded area around ErbB4 KO and Ctrl lines represent the SEM. ***B***, Moreover, an independent cohort of 90-d-old ErbB4 KO and Ctrl mice (*n* = 13/genotype) with free access to food and water were analyzed during 10 consecutive days for body weight, (***C***) energy intake, (***D***) energy expenditure, and (***E***) body mass composition. In agreement to ***A***, 90-d-old ErbB4 KO and Ctrl mice have similar body weights (*p* > 0.05; ***B***). Consistently, the energy intake between ErbB4 KO and Ctrl mice (*p* > 0.05; ***C***) and energy expenditure did not show differences (*p* > 0.05; ***D***). ***E***, Finally, no differences between fat/lean body mass composition was observed between genotypes (*p* > 0.05). Data are expressed as the mean ± SEM.

Given the apparent augmented willingness of ErbB4 KOs to work for palatable food under operant conditions, we considered whether these differences were primarily associated with intrinsic body metabolic requirements or, whether they were mediated by elevated motivation or appetite for palatable food. Accordingly, we initially monitored the body weight of ErbB4 KO and Ctrl mice (*n* = 7/genotype) provided with regular chow and water *ad libitum* in their home cage every 30 d for a full year, and we found no differences between genotypes (*p* > 0.05; Fig. 6*A*). In separate cohorts of mice (*n* = 13/genotype), we then compared the energy balance (intake vs expenditure) and body mass composition (fat vs lean mass) of ErbB4 KOs and Ctrls for two consecutive weeks. Consistent with the previous observation (Fig. 6*A*), daily measurements of their body weight (ErbB4 KO 28.7 ± 2.1 g, Ctrl 26.8 ± 1.4 g, *p* > 0.05; Fig. 6*B*), energy intake (ErbB4 KO 43.3 ± 3.9 kcal/BW, Ctrl 39.3 ± 2.5 kcal/BW, *p* > 0.05; Fig. 6*C*) and expenditure (ErbB4 KO 44.9 ± 3.2 kcal/BW, Ctrl 41.8 ± 2.1 kcal/BW, *p* > 0.05; Fig. 6*D*), and body mass composition (fat mass: ErbB4 KO 3.3 ± 0.7 g, Ctrl 3.3 ± 0.5 g, *p* > 0.05; lean mass: ErbB4 KO 24.2 ± 1.6 g, Ctrl 23.1 ± 1.1 g, *p* > 0.05; Fig. 6*E*) did not uncover differences between genotypes. To the extent assessed here, our data suggest that ErbB4 KOs and Ctrl mice do not show differences in their energy balance and body composition.

## Discussion

Despite the emerging interest in NRG-ErbB signaling in DA function, how mutation of ErbB4 affects tonic DA levels in distinct brain areas and its association with behavioral alterations has remained mostly unknown. Here, we have demonstrated that ErbB4 KOs manifest an imbalance of steady-state extracellular DA across nigrostriatal and meso-cortico-limbic systems. Moreover, we confirm and expand on behavioral deficits observed in ErbB4 KOs relevant to psychiatric disorders, including increased locomotor activity, cognitive-related impairments, and elevated motivation/willingness to retrieve food rewards.

### ErbB4 deletion alters DA homeostasis across nigrostriatal and mesocorticolimbic systems

In the present study we report that ErbB4 KO mice have elevated tonic extracellular DA levels in the dorsal striatum, whereas they have reduced DA levels in mPFC, dorsal hippocampus, and NAc. These changes in tonic DA levels are similar to those observed in mice with targeted mutation of NRG2, a major ErbB ligand in the adult mouse brain (Yan et al., 2018). Interestingly, modulating ErbB receptor activity during neonatal development by systemically administering either NRG1 or a pan-ErbB inhibitor (JNJ-28871063) later cause alterations in DA levels in the adult (Kato et al., 2011; Mizuno et al., 2013; Golani et al., 2014). Moreover, it has recently been reported that targeted mutation of ErbB4 in tyrosine hydroxylase (TH) neurons, but not in PV+ GABAergic interneurons, have decreased striatal and elevated mPFC-hippocampus tonic extracellular DA levels relative to controls (Skirzewski et al., 2018), a DA imbalance pattern that is inversed compared with the ErbB4 KOs described herein. Although we presently do not understand the mechanisms that account for the reciprocal changes of extracellular DA levels across brain regions observed in the pharmacological models, ErbB4 KO, NRG2 KO, and TH-targeted ErbB4 KO mice, these findings suggest that alterations in NRG/ErbB4 signaling can bidirectionally affect the tonic levels of extracellular DA by affecting the activity of DAT in monoaminergic neurons (Skirzewski et al., 2018) and by altering the activity of local microcircuits. Recent retrograde and anterograde tracing studies, coupled to single-cell RNAseq analysis, have uncovered a previously unappreciated complexity of DA neurons that comprise nigrostriatal, mesocortical and mesolimbic connections (Morales and Root, 2014; Lammel et al., 2015; Menegas et al., 2015; Barker et al., 2016; Farassat et al., 2019). An interesting possibility that may account for the different effects of ErbB activity on tonic DA levels, is that NRG/ErbB4 signaling differs among distinct DA neuron populations. There is also evidence that mesocortico/limbic and nigrostriatal DA projections are mutually connected with cortical microcircuitry involving inhibitory GABAergic activity, particularly PV+ fast-spiking interneurons (Uhlhaas and Singer, 2010; Lewis et al., 2011), that can regulate local microcircuits and levels of DA release. An interesting and important aspect of our findings of DA imbalance in ErbB4 and NRG2 KO mice, regardless of the underlying mechanisms, is that they are reminiscent of the DA imbalance reported in schizophrenia patients. In particular, our results recapitulate the hyperdopaminergic state in the striatum and the hypodopaminergic in the DLPFC often reported in schizophrenic patients (Weinstein et al., 2017). This is particularly critical, as it may suggest that the ineffectiveness of D2 receptor-targeting antipsychotics in alleviating cognitive deficits could be associated with reduced neocortical dopaminergic function. Therefore, targeting NRG and ErbB4 mutations in mice to distinct neuronal subpopulations could provide an important model to uncover novel cellular targets and circuits that modulate DA levels in the neocortex, and that are necessary for optimal performance in cognitive tasks.

### ErbB4 is relevant to regulate cognitive function

The findings presented here and earlier studies emphasize the importance of NRG/ErbB4 signaling for cognitive functions (Wen et al., 2010; Shamir et al., 2012; Loos et al., 2014, 2016; Marchisella et al., 2018; Skirzewski et al., 2018; Yan et al., 2018). Specifically, we found that ErbB4 KO mice show behavioral deficits that suggest impairments in declarative (NOR; Fig. 3*A*), spatial (Barnes maze; Fig. 3*C*,*D*), contextual (CPP; Fig. 4), and working memory functions (T-maze; Fig. 3*B*). Previous reports have shown that the medial temporal lobe system, which includes the hippocampus and the perirhinal, entorhinal, and parahippocampal cortices, is necessary to temporarily store factual information as declarative memory and is therefore relevant to encode locations and place-object associations (Squire et al., 2004). Moreover, these structures exhibit a widespread and reciprocal network connectivity with the neocortex to encode, maintain and retrieve relevant information necessary for spatial and working memory (Vann and Albasser, 2011; Spellman et al., 2015; Tamura et al., 2017). Lastly, cortical and hippocampal networks are regulated by GABAergic PV+ interneuron activity (Lewis et al., 2012; Gonzalez-Burgos et al., 2015; Dienel and Lewis, 2019) and are modulated by DA to regulate cognitive function (Williams and Goldman-Rakic, 1995; Goldman-Rakic et al., 2000; Winterer and Weinberger, 2004; Gonzalez-Burgos et al., 2005; Spellman et al., 2015; Cassidy et al., 2016).

Although we cannot establish a causal relationship between the cognitive deficits observed in ErbB4 KOs and the reductions in the relative levels of tonic extracellular DA in the mPFC and hippocampus (Fig. 1), it is tempting to speculate that these reduced levels of DA could contribute to the observed deficits in declarative, spatial, and working memory. It is well documented that DA plays an important role in modulating cognitive functions in primates (Arnsten et al., 2015), and DA receptor-targeting drugs are known to ameliorate acute motor and cognitive deficits in rodents with disruptions in the NRG-ErbB4 pathway (Sotoyama et al., 2011; Andersson et al., 2012; Yan et al., 2018). Reduced levels of DA in both mPFC and dorsal hippocampus of ErbB4 KOs could contribute to the observed deficits in declarative, spatial, and working memory. Interestingly, mice with targeted mutations of ErbB4 in TH+ midbrain neurons manifest a relative increase of tonic extracellular DA levels in mPFC and dorsal hippocampus and also exhibit spatial and working memory deficits (Skirzewski et al., 2018). Of significance, targeted re-expression of ErbB4 in TH positive midbrain neurons of adult TH-ErbB4 KO mice resulted in a recovery of tonic extracellular DA levels and improvement in performance of spatial and working memory tasks (Skirzewski et al., 2018), consistent with an important association between optimal DA levels and cognitive performance.

However, one cannot rule out that other neurons also contribute to the association between ErbB4 and cognitive performance. Of particular relevance is the expression of ErbB4 in mPFC and hippocampal PV+ GABAergic interneurons, which express the highest relative levels of receptor and regulate γ oscillation power (Vullhorst et al., 2009; Fazzari et al., 2010; Neddens et al., 2011). Mice with targeted mutation of ErbB4 in PV+ interneurons manifest working/spatial memory impairments (Wen et al., 2010; Del Pino et al., 2013), but they do not exhibit any changes in tonic extracellular DA levels in the neocortex or hippocampus (Skirzewski et al., 2018). Taken together, these results suggest that NRG-ErbB4 signaling in TH+ neurons and PV+ interneurons both contribute to sustain an optimal local network activity necessary for cognitive function in mice. Further analysis is warranted to better understand how the interaction between tonic extracellular DA levels and neuronal network activity, potentially mediated by cross-talk between DA D4 receptor and ErbB4 receptors on PV+ interneurons (Kwon et al., 2008; Andersson et al., 2012), regulate cognitive performance.

### ErbB4 regulates motivation for palatable food rewards

The NAc is known as the limbic-motor interface in the central nervous system due to its role in integrating synaptic inputs from several brain regions to regulate goal-directed behaviors, motivation and reward (Mogenson et al., 1980; Groenewegen et al., 1999; Goto and Grace, 2008; Averbeck and Costa, 2017; Wise and McDevitt, 2018). Recent studies suggest that abnormal dopaminergic and GABAergic transmission in the NAc of rodents is associated with alterations in motivational functions (Stratford and Kelley, 1997; Reynolds and Berridge, 2001; Peciña et al., 2003; Rada et al., 2010; Smith et al., 2011; Beeler et al., 2012; Soares-Cunha et al., 2018), such as the exertion of effort during instrumental behavior, flexible approach behavior and exploitation of reward learning (Peciña et al., 2003; Rada et al., 2010; Smith et al., 2011; Beeler et al., 2012). As reported here, ErbB4 KO mice are more motivated than Ctrls to work for palatable food rewards by mechanisms unrelated to altered energy balance (Figs. 5, 6). Consistent with previous reports using systemic administration of JNJ28871063 (Golani et al., 2014; Tadmor et al., 2017, 2018), our findings show that NRG-ErbB4 signaling affects circuits that modulate reward-related behaviors. The regulation of motivational behaviors by NRG-ErbB4 signaling could potentially be attributed to changes in extracellular DA levels (this study) or to GABAergic transmission (Geng et al., 2017). A future challenge will be to identify the ErbB4-dependent mechanisms in the NAc and associated networks that drive this increased motivation to work for palatable food rewards.

### Concluding remarks

This and prior reports have shown that the NRG-ErbB4 signaling modulates the dopaminergic and GABAergic neurotransmission systems. The functional interaction between these signaling pathways can contribute to regulate E/I balance, synaptic plasticity, synchrony network activity and phenotypes relevant to psychiatric disorders (Law et al., 2007; Buonanno, 2010; Kao et al., 2010; Greenwood et al., 2012; Joshi et al., 2014; Mei and Nave, 2014; Mostaid et al., 2016). Moreover, because the NRG-ErbB4 signaling pathway functionally interacts to regulate synaptic and network properties underlying complex behavioral traits, our findings highlight why classical therapeutic approaches targeting specific neurotransmitter pathways to treat the diverse symptoms in psychiatric disorders, including schizophrenia, have had relatively little success.

## Synthesis

Reviewing Editor: Karen Szumlinski, University of California at Santa Barbara Decisions are customarily a result of the Reviewing Editor and the peer reviewers coming together and discussing their recommendations until a consensus is reached. When revisions are invited, a fact-based synthesis statement explaining their decision and outlining what is needed to prepare a revision will be listed below. The following reviewer(s) agreed to reveal their identity: Stephanie Groman.

Upon consultation with two reviewers with expertise in the neuropharmacology and neurophysiology of the dopamine system, we have reached the decision of “Revise and Re-review” on your report concerning the phenotype of ErbB4 KO mice. Both reviewers and I felt that the data presented were novel and impactful, as they provided a very thorough characterization of the consequences of deleting ErbB4 for both subregional regulation of basal dopamine content and for behavior. This being said, both reviewers raised some concerns regarding the manuscript as currently written but we all agree tha these revisions should require less than 2 months’ work. In particular, a major concern relates to the assumption that the behavioral phenotype of the mice relates directly to perturbations in extracellular dopamine. Cause-effect evidence for such a relationship is critical to tying the data sets together, particularly in light of published work on mice with selective ErbB4 deletion from TH+ neurons. All three of us have issue with the lack of methodological detail provided concerning the behavioral test battery and request that the experimental designs be more carefully explained, particularly if the same animals were employed in behavioral and microdialysis studies. Both reviewers also raised some additional issues, and I detected several typos and grammatical errors in the Significance Statement, as well as throughout the text, that should also be addressed in the revision. Please find the specific comments and queries raised by the reviewers below:

Reviewer 1:

The manuscript titled ‘Dysfunctional dopamine system in ErbB4 Null mice is associated with cognitive and motivational behavioral deficits’ describes a study examining how genetic deletion of ErbB4 affects dopamine and behavioral processes that are thought to model the behavioral impairments observed in individuals with schizophrenia. The authors collected microdialysis samples from four different brain regions to quantify dopamine and dopamine related metabolites in control and ErbB4 KO mice. They report that dopamine tone is lower in in the ventral striatum, mPFC and hippocampus in ErbB4 KO mice compared to controls, but increased in the dorsal striatum. They also assessed behavior in these mice using a wide range of laboratory tasks (Barnes maze, food-based CPP, novel object recognition, T maze, etc) and found divergent effects between ErbB4 KO and controls that may be linked to the altered dopaminergic signaling reported in this paper. The paper and experiments are well written and the results are very interesting and will likely be very valuable as a reference for other studies examining the role of ErbB4 in psychiatric disorders. I have a few comments for the authors that are outlined below that I believe if addressed could enhance the impact of the manuscript.

1. It is not clear why the role of Erb4 in visual system development is mentioned in the significance statement as none of the experiments examined visual system development. It’s fine to leave in the introduction, but I don’t think it’s necessary to include in the significance statement.

2. The authors state that 4 cohorts of mice were used for 12 different behavioral tasks, so presumably some mice were assessed on multiple behavioral tasks. The authors should outline which mice were repeatedly used - for example, did the amphetamine exposed mice then do the T maze? Did the Barnes maze mice then do Pavlovian task? The order of training could impact performance across tasks.

3. The dose of amphetamine is described as nM in the text, but then reported as mg/kg in the figures. Please change text to mg/kg as this is standard reporting for pharmacological experiments.

4. The results are very interesting, but difficult to interpret. I think it would be beneficial if the authors provided a more cohesive and comprehensive discussion about why global deletion of ErbB4 would have such divergent behavioral and biological effects. Does ErbB4 expression normally differ in the meso-striatal compared to the meso-cortical circuits? Is this receptor expressed on different cell types in these regions that could explain why genetic deletion results in divergent anatomical and behavioral differences?

5. The logic for the use of the multiple behavioral tasks is well laid out in the results, but then is described in the discussion collectively as being ‘cognitive-related’ behaviors. I think the authors should be more specific about their findings in the discussion, rather than grouping the results together as cognitive deficits. In fact, it seems to me that the behavioral results indicate that ErbB4 may play a role in memory consolidation, which the authors might consider as a discussion point.

Reviewer 2

Comments to the Authors (Required) This work investigates the baseline (tonic) dopamine levels and the behavioral phenotype of mice deficient for the Neuregulin receptor ErbB4 (ErbB4 KO mice). Using microdialysis/HPLC detection, the authors report an imbalanced dopamine tone in ErbB4 mice compared to wild-type controls (elevated in dorsal striatum, but reduced in NAc, mPFC and Hippocampus). Using a relatively large battery of behavioral tasks, the authors also report overall reduced spatial memory, but increased sensitivity to amphetamine and increased motivation to work for reward in ErbB4 mice.

This work has important implication for the understanding of the genetic linkage between the Neuregulin-ErbB receptor (NRG-ErbB) signaling pathway and schizophrenia. Generally speaking, data are presented in a manner that is easy to understand, appropriate controls have been run, appropriate statistical analyses have been conducted, and the manuscript is well written.

Main points:

1) My main criticism concerns the causal relationship that the authors seem to draw from the dopamine level measurements and the behavioral performances observed in ErbB4 mice. Indeed, in the text -and the general structure of the manuscript- the authors strongly imply/presume a causal relationship between the altered level of dopamine in a given brain region, and the behavioral performance in a given task (ex: dorsal striatum dopamine presumably explains performance on open field; mPFC and Hippocampus dopamine presumably explains T-maze performance; NAc dopamine presumably explains CPP performance, etc.). Although dopamine transmission in those regions has indeed been shown (in previous studies) to influence performance in these tasks, it doesn’t necessarily mean that in this study -in those ErbB4 KO mice- the behavioral deficits observed in a given task are due to the increase/decreased dopamine tone in a given brain region. As mentioned by the authors in the introduction, ErbB4 deletion affects several cell types and neural circuits, beyond dopamine (cortical, hippocampal, or amygdala GABA interneurons development and/or activity), which could contribute to the behavioral phenotype observed in those mice. This work made no attempt to further demonstrate the causal role of dopamine imbalance observed in ErbB4 KO mice and the behavioral phenotype of these mice. Admittedly, this point is mentioned briefly in the discussion, but this should be highlighted/further discussed. Moreover, to avoid the presumption of an association between dopamine tone and behavioral results, the authors might want to consider changing the structure of the results section, (ex: present all microdialysis results, followed by all behavioral results), and then use the Discussion to discuss the possible causal relationship between dopamine and behavior.

2) Authors report increased striatal and decreased cortical and hippocampal dopamine levels in ErbB4 KO mice. In contrast, previous studies in mice lacking ErbB4 specifically in TH+ neurons showed the mirror opposite: decreased striatal and elevated cortical and hippocampal dopamine. Can the authors comment on this?

3) P15, Line 344: The authors wrote that “DA levels in cortical and hippocampal regions of ErbB4 KOs correlate with spatial learning and memory deficits”. However, no correlation analysis is provided, and based on the method section, no correlation analysis is likely possible here (dialysate pooled from 2 mice). But what about the other brain regions (NAc and dorsomedial striatum)? Have the authors attempted to run a correlation analysis of these measures (DA levels / behavioral performance)?

4) Related to point 2: were the same animals used for microdialysis and behavioral tests? This should be clarified in the methods.

5) Were the same animals subjected to more than one behavioral assay? If so, please specify the different tests and the order.

Minor comments:

P2, line 32: “resembles what repeatedly reported in schizophrenia patients"

P5, line 104: “At least otherwise indicated"
